# Nanocarrier-enhanced intracellular delivery of benznidazole for treatment of *Trypanosoma cruzi* infection

**DOI:** 10.1172/jci.insight.145523

**Published:** 2021-05-10

**Authors:** Xiaomo Li, Sijia Yi, Débora B. Scariot, Santiago J. Martinez, Ben A. Falk, Cheryl L. Olson, Patricia S. Romano, Evan A. Scott, David M. Engman

**Affiliations:** 1 Department of Pathology and Laboratory Medicine, Cedars-Sinai Medical Center, Los Angeles, California, USA.; 2 Department of Pathology, Northwestern University, Chicago, Illinois, USA.; 3 Department of Biomedical Engineering, Chemistry of Life Processes Institute, and Simpson Querrey Institute, Northwestern University, Evanston and Chicago, Illinois, USA.; 4 Institute of Histology and Embryology, “Dr. Mario H. Burgos”, IHEM-CONICET, National University of Cuyo, Mendoza, Argentina.; 5 Department of Pathology and Laboratory Medicine, University of California, Los Angeles, Los Angeles, California, USA.

**Keywords:** Microbiology, Drug therapy, Parasitology

## Abstract

Chagas disease is caused by infection with the protozoan parasite *Trypanosoma cruzi* (*T*. *cruzi*), an intracellular pathogen that causes significant morbidity and death among millions in the Americas from Canada to Argentina. Current therapy involves oral administration of the nitroimidazole benznidazole (BNZ), which has serious side effects that often necessitate cessation of treatment. To both avoid off-target side effects and reduce the necessary dosage of BNZ, we packaged the drug within poly(ethylene glycol)-block-poly(propylene sulfide) polymersomes (BNZ-PSs). We show that these vesicular nanocarriers enhanced intracellular delivery to phagocytic cells and tested this formulation in a mouse model of *T*. *cruzi* infection. BNZ-PS is not only nontoxic but also significantly more potent than free BNZ, effectively reducing parasitemia, intracellular infection, and tissue parasitosis at a 466-fold lower dose of BNZ. We conclude that BNZ-PS was superior to BNZ for treatment of *T*. *cruzi* infection in mice and that further modifications of this nanocarrier formulation could lead to a wide range of custom controlled delivery applications for improved treatment of Chagas disease in humans.

## Introduction

Chagas disease is an insect-transmitted parasitic infection first described in 1909 by the Brazilian physician Carlos Chagas (1). The protozoan *Trypanosoma cruzi* (*T*. *cruzi*) causes lifelong infection in humans and other vertebrates by infecting a wide variety of cells throughout the body, typically controlled by adaptive immunity without causing any of the adverse sequelae known as Chagas disease. Indeed, most *T*. *cruzi*–infected individuals are unaware of their infections, with approximately one-third developing either a chronic cardiomyopathy or a megadisease of the esophagus or colon, often many years after infection (2). There are approximately 7 million infections and 14,000 deaths each year from *T*. *cruzi* and Chagas disease worldwide (3), and several hundred thousand infected people currently reside in the United States (4). Although most *T*. *cruzi*–infected people in the United States are immigrants who were infected in their countries of origin, *T*. *cruzi*–infected triatomine bugs are found in most Southern states, and vector-borne autochthonous transmission is now understood to be a small but significant risk to many Americans (5). Blood transfusion and organ transplantation are additional modes of transmission, but these have been reduced significantly through global screening of blood and organs for *T*. *cruzi* (6).

Only 2 drugs, the hydrophobic nitroimidazoles benznidazole (BNZ) and nifurtimox (NFX), have been available to treat *T*. *cruzi*–infected individuals since 1970 (7). Despite their poor bioavailability and permeability (8), both drugs are up to 80% effective when used during acute infection. Unfortunately, these agents have severe side effects, including neutropenia, nausea, vomiting and diarrhea, weight loss, and hypersensitivity skin reactions and hives, which lead to treatment cessation in many individuals (9). In chronic disease, when cardiomyopathy and megadisease typically develop, the decision to treat has been a matter of debate for many years. The very large BENEFIT trial tested the effect of treatment on those with established cardiomyopathy and concluded that treatment does not reduce progression to a major cardiac event or death, despite reducing parasitemia (10). However, results from a more recent BNZ clinical trial showed that treatment of indeterminate individuals having no clinical disease reduces progression to cardiomyopathy (11), which many consider equally important. Regardless, there is no treatment that reverses Chagas cardiomyopathy once established (12). Chagas is considered a neglected tropical disease and is still overlooked by pharmaceutical companies due to it primarily being found within low-income countries (13, 14).

*T. cruzi* is an obligate intracellular pathogen that can infect any nucleated cell. The tropism to myocytes has been extensively studied because the presence of parasite in muscles cells is intrinsically related to cardiac dysfunction, especially in the acute stage (2). Therefore, trypanocidal drugs must have the ability to cross the host cell plasma membrane to effectively kill intracellular parasites (14). It is possible that the toxicity observed with BNZ and NFX treatment is due to the high concentration necessary to achieve an effective intracellular killing concentration. The development of drug nanocarriers has revolutionized therapeutics by permitting drug transport to specific organs, cells, and intracellular targets, thus reducing toxicity and enhancing potency (15, 16). Whereas the development of these delivery platforms has focused mainly on the treatment of cancer (17, 18), several studies have presented promising results after the delivery of BNZ and NFX via nanocarriers (13, 19, 20).

BNZ nanocrystals produced by a nanoprecipitation process have been shown to improve the BNZ solubility and permeability, potentiating BNZ trypanocidal activity in acute Chagas disease despite the nanocrystal instability (21). Mesoporous silica nanoparticles combined with chitosan facilitates cellular uptake, which enhances in vitro trypanocidal activity, but with significant cytotoxicity (22). Complexes of BNZ and cyclodextrin have been reported as a strategy to increase the hydrophilicity of BNZ, which improves the absorption, permeability, and bioavailability of BNZ oral formulations. Modifying these pharmacokinetic properties reduces the toxicity of BNZ, possibly by reducing lipophilicity of the drug (23) without inhibiting trypanocidal activity (24). Nevertheless, the BNZ-cyclodextrin complex was not superior to free BNZ in trypanocidal activity during murine *T*. *cruzi* infection (25). BNZ liposomes have also been investigated due to their biocompatibility and biodegradability. However, their rapid hepatic clearance and low BNZ encapsulation efficiency are likely responsible for suboptimal in vivo efficacy (25, 26). Finally, incorporation of BNZ into a self-emulsifying delivery system has been successfully used in children (27).

Mice represent an excellent animal model of human Chagas disease, with different parasite-mouse and strain-strain combinations being able to represent diverse outcomes of infection, ranging from no disease to acute fulminating disease and death. In the present study, we developed BNZ-loaded vesicular nanocarriers by packaging BNZ within poly(ethylene glycol)-block-poly(propylene sulfide) (PEG-*b*-PPS) polymersomes (BNZ-PSs), which we compared with free BNZ in a mouse model of *T*. *cruzi* infection. PEG-*b*-PPS nanocarriers have been employed for enhanced delivery and efficacy with lower toxicity for a wide range of therapeutic and diagnostic agents (28–30).

## Results

### Production and characterization of BNZ-PSs.

**Figure 1:**
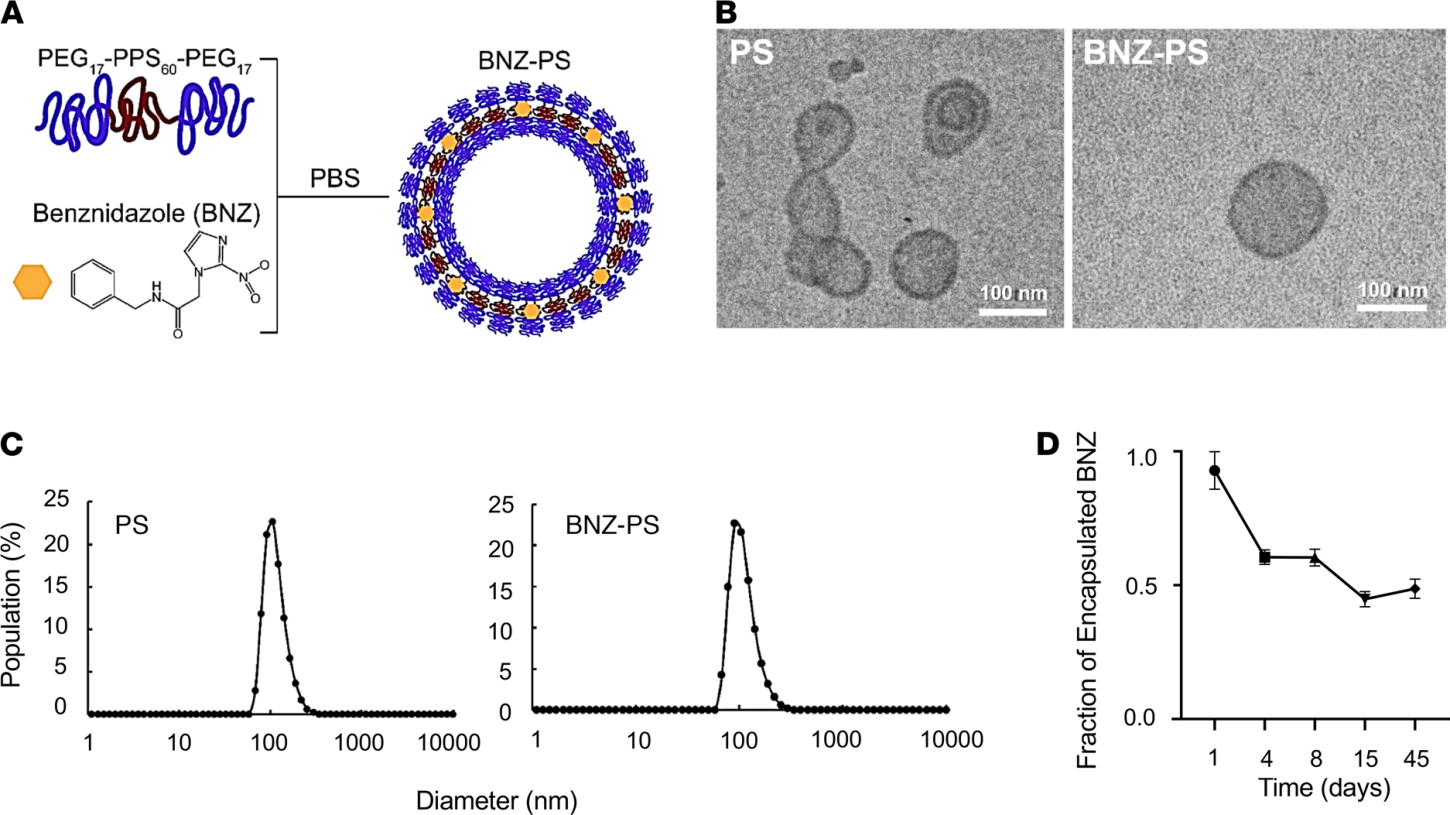
BNZ-PSs are 100 nm particles with good in vitro stability. (**A**) BNZ was loaded into PSs by the thin-film hydration method. (**B**) Representative cryo–transmission electron microscopy images of PS and BNZ-PS. Scale bar: 100 nm. (**C**) The diameters of PS and BNZ-PS are approximately 100 nm as determined by dynamic light scattering analysis. (**D**) The stability of BNZ-PS was determined by incubating the particles in PBS at 4°C and quantifying the BNZ in BNZ-PS over time by LC-MS. Results expressed as mean ± SEM for 3 independent experiments. LC-MS, liquid chromatography–coupled mass spectrometry.

**Figure 2:**
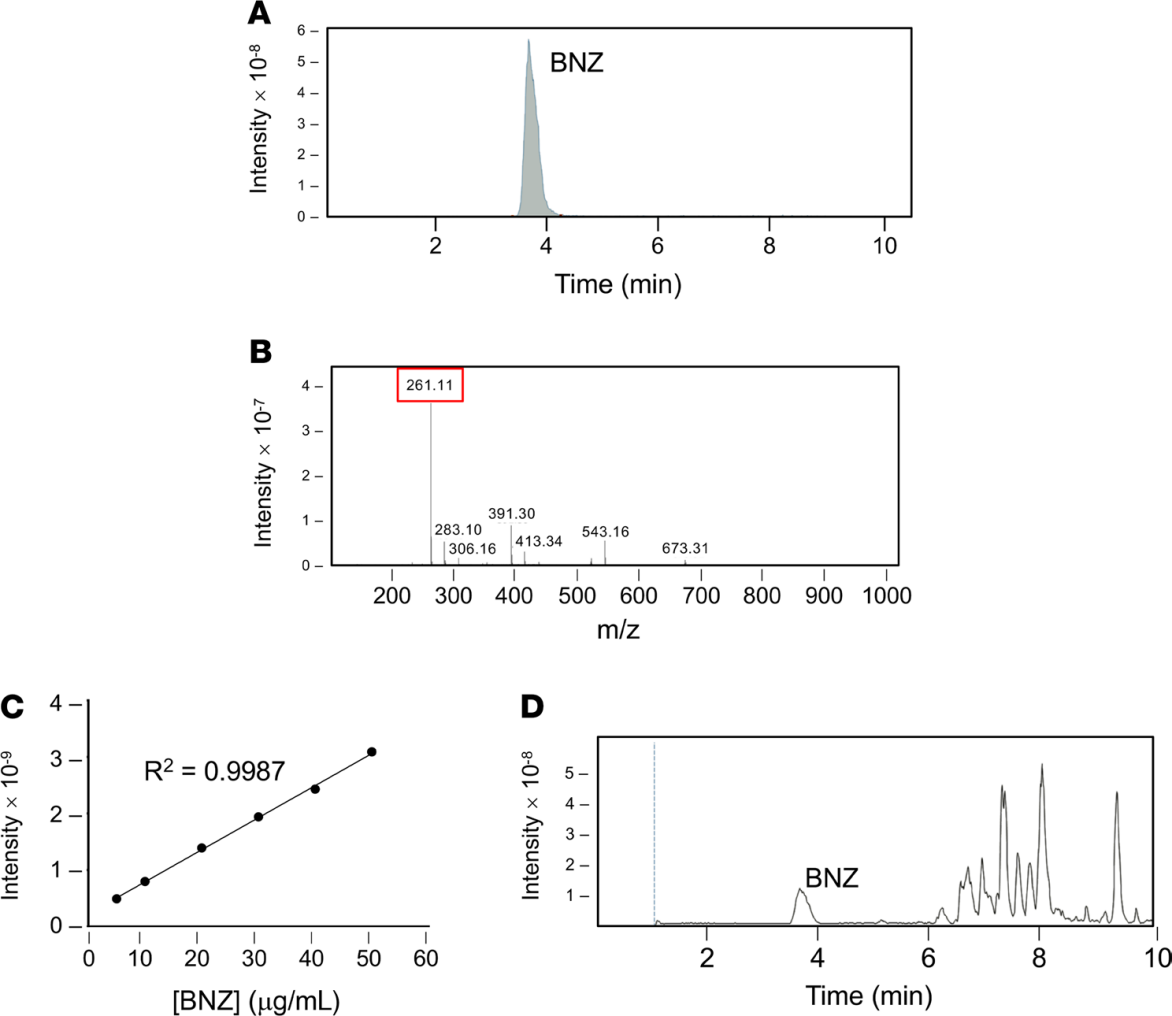
Analysis of BNZ loading within BNZ-PSs. (**A**) Representative chromatogram and (**B**) positive ion mass spectrum of BNZ standards. The BNZ peak is indicated at *m/z* of approximately 261. (**C**) Calibration curve of BNZ standards using ordinary least square. (**D**) Representative chromatogram of BNZ-PS displaying a BNZ peak at the same elution time as BNZ standards in **A**.

BNZ-PSs were prepared using the thin-film hydration method (Figure 1A). BNZ-PS and unloaded PS have similar structures and sizes, as determined by cryogenic transmission electron microscopy (Figure 1B) and dynamic light scattering (Figure 1C), respectively. The hydrodynamic size of BNZ-PS was approximately 115 nm, which is comparable to unloaded PS (Table 1). Although PEG-*b*-PPS PS is effectively neutral in surface charge (zeta potential), that of BNZ-PS in PBS is slightly positive. The encapsulation efficiency and loading efficiency of BNZ-PS were approximately 31% and approximately 1%, respectively (Table 1), and stable for well over a month at room temperature (Figure 1D). LC-MS was used to characterize BNZ loading within PEG-*b*-PPS PS (Figure 2).

### Effective killing of human life cycle stages of T. cruzi by BNZ-PSs.

**Figure 3:**
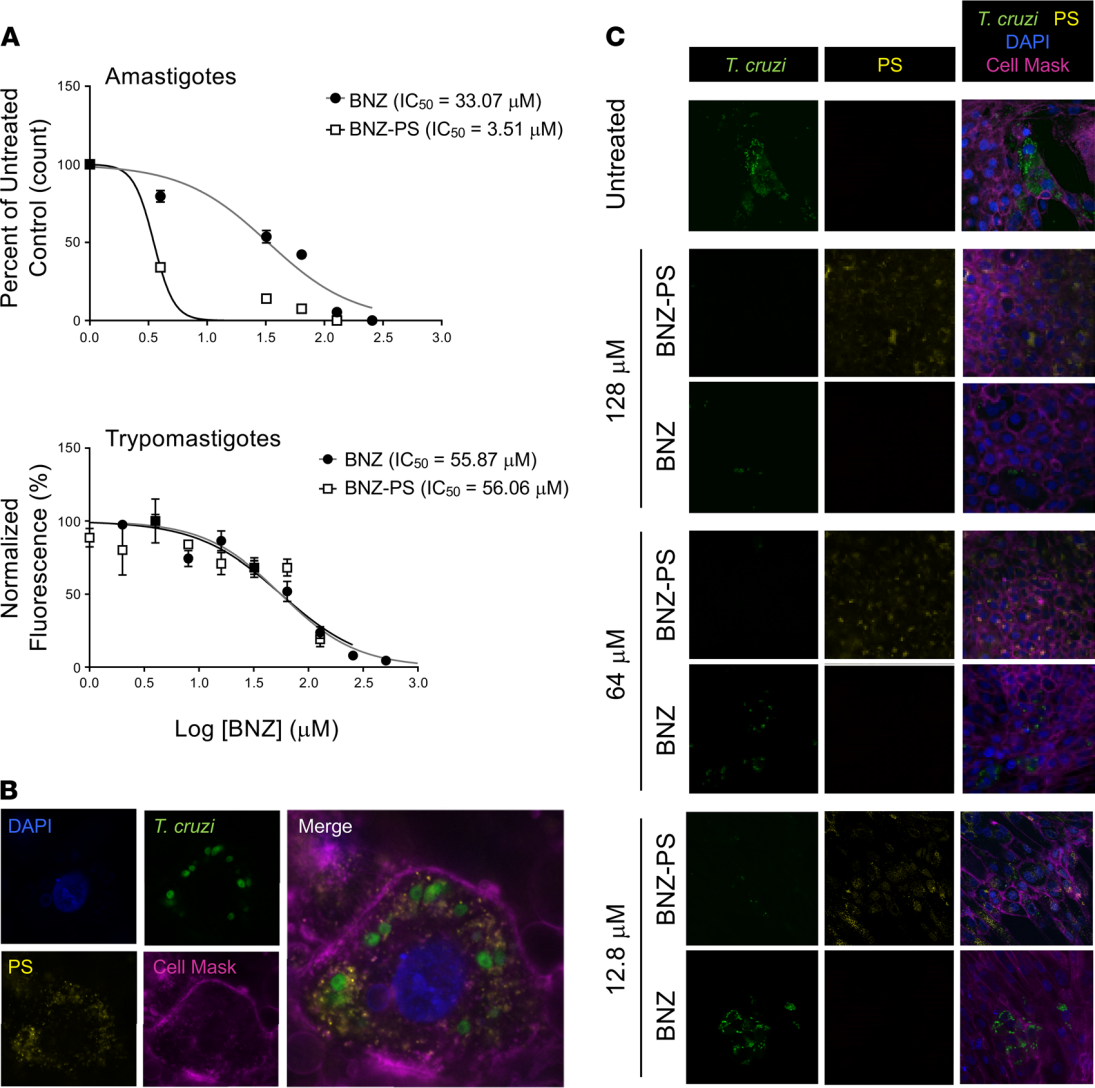
BNZ-PSs are more potent than free BNZ against *T. cruzi* in vitro. (**A**) In vitro killing of purified *T*. *cruzi* Y strain trypomastigotes by BNZ and BNZ-PS. Trypomastigotes were purified from infected H9C2 cell cultures and tested in a 24-hour resazurin cell viability assay using increasing doses of BNZ or BNZ-PS. Results from 3 independent experiments are expressed as mean ± SEM. (**B**) PS are readily taken up by *T*. *cruzi*–infected H9C2 cells. H9C2 cells were infected with *T*. *cruzi* Tulahuen strain trypomastigotes expressing Luc-mNeonGreen (green) for 24 hours and Alexa Fluor 630–labeled PS (yellow) were added and cultures incubated for an additional 24 hours. Cells were imaged after staining with DAPI (blue) and Cell Mask Deep Red Dye (purple) (original magnification, ×1000). (**C**) BNZ-PSs are significantly more potent against intracellular *T*. *cruzi* than free BNZ. Cells were cultured and treated as in **B**, but with different concentrations of BNZ or BNZ-PS, and imaged after DAPI and Cell Mask staining. The key images for comparison are the left (*T*. *cruzi*) BNZ and BNZ-PS panels at each drug concentration (original magnification, ×400).

The trypanocidal effectiveness of BNZ-PSs was evaluated in vitro against 2 human life cycle stages, the amastigote form, which replicates in the host cell cytoplasm, and the trypomastigote form, which arises from the amastigote through differentiation and is liberated from the host cell as it lyses to infect adjacent cells or distant cells after hematogenous spread. The half-inhibitory concentrations (IC_50_) of free BNZ and BNZ-PSs against amastigotes were 33.07 ± 8.17 μM and 3.51 ± 0.79 μM, respectively, and against trypomastigotes were 55.87 ± 11.39 μM and 56.06 ± 12.21 μM, respectively (Figure 3A). Therefore, although the 2 drug preparations were similarly effective against free trypomastigotes, BNZ-PSs were effective at one-tenth the concentration of free BNZ against amastigotes. The spatial relationships among the host cell, intracellular *T*. *cruzi* amastigotes, and Alexa Fluor 630–loaded PSs are easily seen (Figure 3B), indicating a successful uptake of PSs by host cells. The effects of including different concentrations of BNZ in the PSs on the levels of *T*. *cruzi* are also clearly visualized (Figure 3C). As the concentration of BNZ/BNZ-PSs was decreased, a clear increase in *T*. *cruzi* was observed in the BNZ-treated cells but not in the BNZ-PS–treated cells. These results clearly demonstrate the superiority of BNZ-PSs to free BNZ at each drug concentration.

### BNZ-PSs are superior to free BNZ in treating T. cruzi–infected mice.

**Figure 4:**
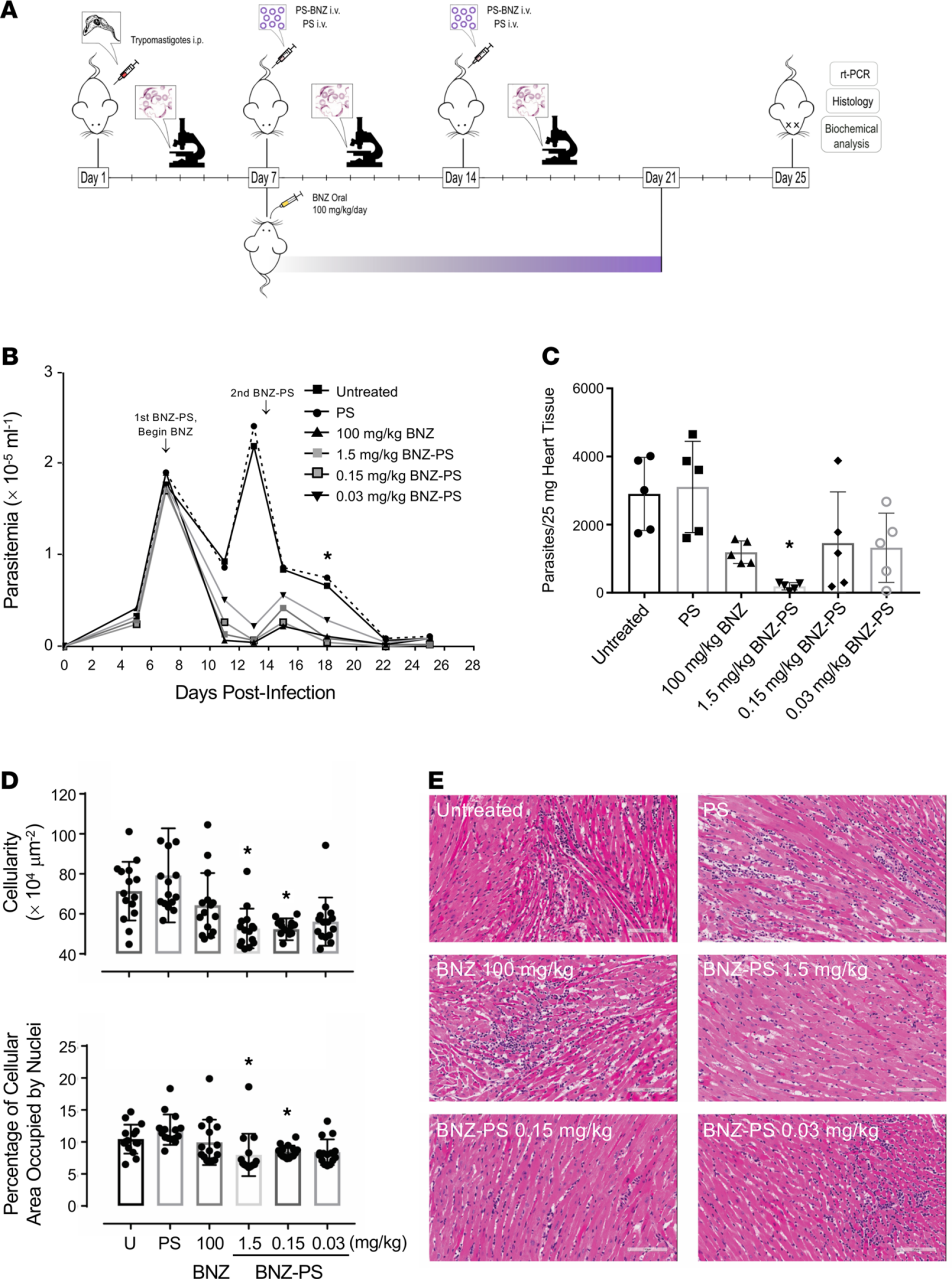
BNZ-PSs are more potent than free BNZ against *T. cruzi* in vivo. (**A**) Mice (*n* = 5 per group) were infected with *T*. *cruzi* Y strain trypomastigotes on day 0 and treated with 2 i.v. doses of BNZ-PS and PS after parasitemia had reached approximately 2 × 10^5^/mL on day 7. Standard oral treatment with BNZ was given daily for 14 days (7–21 d.p.i.). Parasitemia was monitored every few days through the end of the experiment on day 25, when mice were sacrificed and organs were collected for further analysis. (**B**) Effective suppression of parasitemia by BNZ and BNZ-PS. *P ≤ 0.05 for BNZ 100 mg/kg and BNZ-PS 1.5 and 0.15 mg/kg vs. untreated and PS. (**C**) Cardiac parasitosis was quantitated by quantitative PCR. *P ≤ 0.01 vs. untreated and PS. (**D**) Cardiac inflammation was quantitated in heart sections 2 ways — by total cellularity (top) and by the percentage of cellular area occupied by nuclei (bottom). *P ≤ 0.05 vs. untreated, PS, and BNZ. (**E**) Representative cardiac histology from the experiment in **C**. Scale bar: 100 μM. All error bars reflect mean ± SEM. One-way ANOVA with Tukey’s post hoc test was used for multiple comparisons.

We infected BALB/c mice with the Y strain of *T*. *cruzi*, which leads to acute myocarditis and eventual development of chronic cardiomyopathy. We began drug administration at 7 days postinfection (d.p.i.) when parasitemia approached 2 × 10^5^ trypomastigotes per milliliter. Mice were administered no drug, empty PSs, free BNZ at 100 mg/kg, or 3 concentrations of BNZ-PS (Figure 4A). Parasitemia dropped in all groups by 11 d.p.i., as expected in this model of *T*. *cruzi* infection, then rebounded by 11 d.p.i. in the untreated and PS groups. Parasitemias in the treatment groups generally paralleled the BNZ concentrations employed. The development of adaptive immunity caused a drop in parasitemia in all groups by 15 d.p.i., as expected, but by 18 d.p.i. significant differences were observed among the groups, with the BNZ 100 mg/kg and PS 1.5 and PS 0.15 mg/kg groups all having substantially lower parasitemia than the untreated and empty PS controls. The group receiving the extremely low dose of 0.03 mg/kg BNZ-PSs showed a 50% reduction in parasitemia, but the difference from untreated controls did not reach statistical significance. Interestingly, the only treatment that significantly reduced cardiac parasitosis, the number of parasites in the heart, was BNZ-PSs at 1.5 mg/kg (Figure 4B). Even BNZ at 100 mg/kg did not reduce parasitosis any better than the other BNZ-PS formulations. The medium-dose BNZ-PS (0.15 mg/kg) also significantly reduced myocarditis (Figure 4C). Representative cardiac histology is shown (Figure 4E), with hearts from untreated, PS-treated, BNZ 100 mg/kg–treated, and BNZ-PS 0.03 mg/kg–treated mice showing focal to diffuse interstitial inflammation and hearts from BNZ-PS 1.5 and 0.15 mg/kg–treated mice displaying normal cardiac histology (Figure 4D).

### BNZ-PS preparations are not toxic to mice.

**Figure 5:**
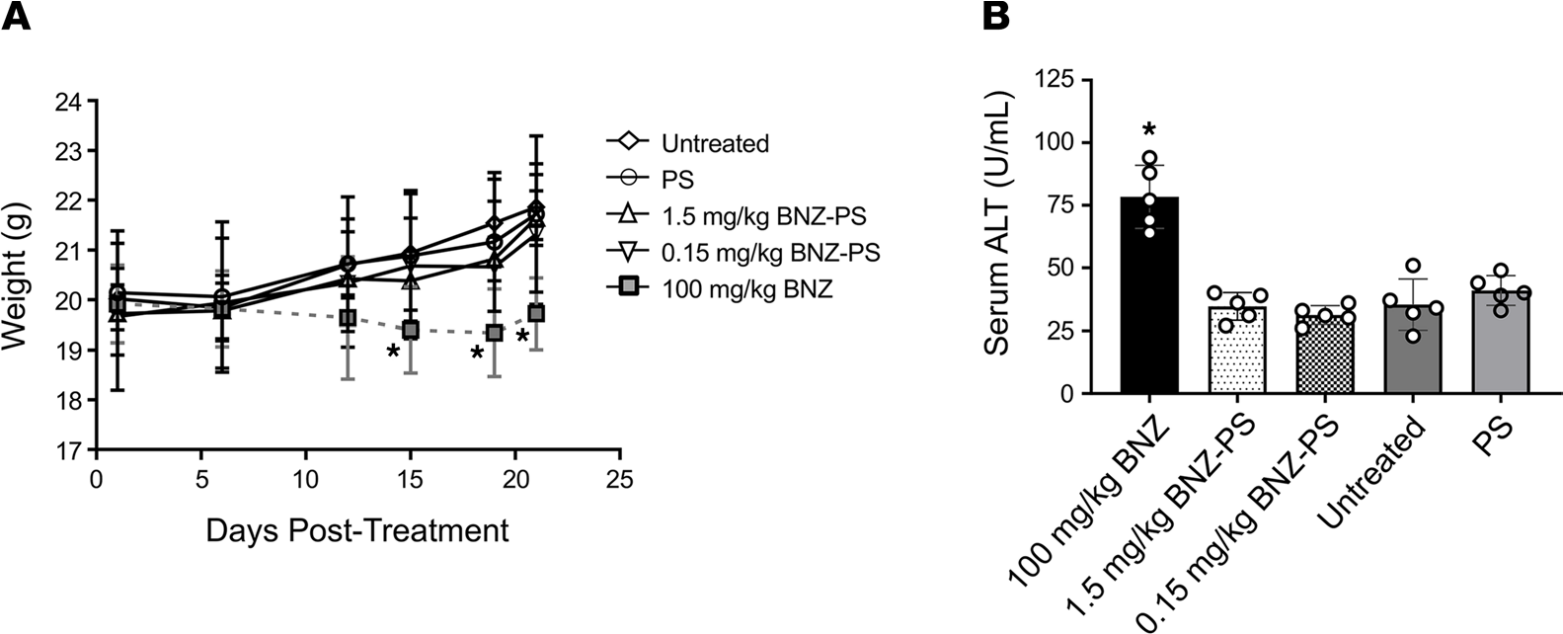
BNZ-PSs are significantly less toxic than free BNZ. Healthy mice (*n* = 5 per group) were treated with BNZ (100 mg/kg) or BNZ-PS (1.5 or 0.15 mg/kg), plus controls. (**A**) Mice treated with BNZ, but not BNZ-PS, lost weight during 2 weeks of treatment. Weights were determined every few days. *P ≤ 0.05 vs. untreated. (**B**) Mice treated with BNZ show hepatotoxicity as reflected by increased serum ALT on day 21. Serum ALT was also measured and did not show significant elevation in any mouse. *P ≤ 0.0001. All error bars reflect mean ± SEM. One-way ANOVA with Tukey’s post hoc test was used for multiple comparisons. ALT, alanine aminotransferase.

To test whether effective doses of BNZ-PSs would also have the benefit of reduced toxicity, groups of mice were given no treatment or were administered PS or doses of BNZ or BNZ-PSs previously determined as effective. Mice treated with BNZ experienced significant weight loss, whereas all other mice gained weight during the 21-day course of the experiment (Figure 5A). BNZ caused a doubling of the serum alanine aminotransferase concentration relative to all other mice (Figure 5B).

## Discussion

A large variety of nanocarriers have been developed for drug delivery that differ in their physicochemical properties, including surface chemistry, shape, size, charge, stiffness, and stability (31, 32). Each of these properties, as well as the molecular payload incorporated, affects biodistribution, cell membrane interactions, mechanisms of cell uptake, and blood protein interactions (30, 33). Predicting how specific nanocarriers influence drug efficacy remains an active area of research, with thousands of possible combinations of physicochemical properties that can be customized for a variety of applications (34). In this regard, PEG-*b*-PPS nanocarriers display high loading efficiency for a wide array of small molecules, increase the effective water solubility of these agents, and enhance intracellular delivery (30, 35–37). They are noninflammatory and nontoxic in mice (38, 39) and nonhuman primates (40). Vesicular nanocarriers like PS display a superior capacity to target antigen-presenting cells, such as DCs and macrophages.

In this study, PEG-*b*-PPS was engineered to assemble vesicles loaded with BNZ, a hydrophobic drug, as the lipophilic PPS block ensures stable BNZ incorporation into the vesicle membrane. The combination of PPS hydrophobicity and PEG hydrophilicity creates a highly stable macroamphiphile copolymer assembly (41). BNZ-PSs are similar in structure and stability to prior PEG-*b*-PPS PS formulations (28–30). We found that incorporation of BNZ into PEG-*b*-PPS PSs reduced by 9-fold the IC_50_ for intracellular *T*. *cruzi* amastigotes, the predominant life cycle stage of the parasite during human infection, while having the same IC_50_ as free-form BNZ for trypomastigotes. The high trypanocidal efficiency of BNZ-PSs for amastigotes is most likely due to the fact that the host cell effectively concentrates BNZ through nanoparticle uptake. Cellular uptake of nanocarriers smaller than 500 nm, such as PEG-*b*-PPS PSs, occurs primarily by endocytosis (42), with cell entry via both macropinocytosis and receptor-mediated endocytosis (30, 37, 39). Once inside the cell, the nanocarrier becomes unstable in the cellular microenvironment (43) with oxidative enzymes inside the PS-containing endolysosomal vacuoles promoting the oxidation of sulfide moieties in the PPS block that modify the hydrophilic-lipophilic balance of the PEG-*b*-PPS copolymer (37). This promotes disassembly of the nanostructure and consequent drug release during the initial stages of oxidation. The vesicle disassembly also liberates amphiphilic copolymers that insert into the endosomal membrane, promoting membrane permeabilization and escape of PEG-*b*-PPS vesicle drug payloads into the cell cytoplasm (36). During cell invasion, *T*. *cruzi* trypomastigotes first enter a parasitophorous vacuole and then escape from the vacuole into the cytoplasm after vacuolar acidification by lysosomal fusion. Based on prior work with PEG-*b*-PPS nanocarriers, the enhanced therapeutic effect of BNZ-PS is likely due to protection of BNZ from the acidic pH of endolysosomal vacuoles and the ability of PEG-*b*-PPS nanocarriers to improve cytoplasmic delivery via nontoxic disruption of endosomal lipid bilayer membranes (37, 44). Thus, the cell invasion and uptake pathways of *T*. *cruzi* and PEG-*b*-PPS are very similar, which may also increase the efficacy of treatment. Two other features of PEG-*b*-PPS are important. First, the copolymer is physiologically inert, releasing drug only after cellular internalization, which may also contribute to the reduced toxicity (45–47). Second, PEG-*b*-PPS enhances the solubility of highly hydrophobic agents like BNZ via entrapment within a lipophilic PPS membrane, allowing more facile and stable transport in the blood, and enhances cellular uptake (36, 38, 39).

The most important finding from our study is that BNZ-PS was extremely potent in treating *T*. *cruzi*–infected mice, with no detectable hepatotoxicity. The total amount of BNZ delivered by 2 injections of BNZ-PS is 466-fold lower than that delivered by daily dosing of free BNZ, leading to similar decreases in parasitemia with notable, if not statistically significant, decreases at even a 23,000-fold lower dose (the cumulative amount over the entire experiment). Although parasitemia may be a useful measure of infection and drug efficacy, the major serious sequela of infection is myocarditis/cardiomyopathy. We assessed the efficacy of the various drug preparations to prevent cardiomyopathy in 2 ways: cardiac parasitosis and cardiac inflammation, with both metrics frequently tracking together. Importantly, BNZ-PSs at 1.5 mg/kg significantly reduced myocarditis, whereas free-form BNZ at 100 mg/kg/d did not. Even at one-tenth the dose (0.15 mg/kg), BNZ-PS reduced cardiac inflammation, although it did not significantly reduce cardiac parasitemia. This finding suggests that BNZ-PSs had the additional benefit of ameliorating cardiac inflammation, even in the presence of a substantial parasite tissue burden. The potential immunomodulatory effect of BNZ-PSs needs further exploration. Although our study was of short duration, possibly representative of acute human infection, the antiinflammatory effect observed here suggests that BNZ-PSs might be effective in chronic Chagas disease, particularly because the same mechanisms of inflammation, immunity, and intracellular parasite killing by drugs are similar in chronic infection.

BNZ is widely used for the treatment of *T*. *cruzi* infection. However, therapeutic efficacy requires the administration of high daily doses of the drug, which is frequently accompanied by toxicity and additional adverse side effects (48, 49). The low BNZ serum half-life in mice (t_1/2_ = 2 h) results from the mouse’s high metabolic rate, including high first-pass metabolism after oral administration with resultant low bioavailability. This is why high BNZ doses of 100 mg/kg/d are required (8). In humans, BNZ displays a longer serum half-life (t_1/2_ = 12 hours) (19) and a lower metabolic rate. Therefore, a lower effective dose is needed but daily doses are still necessary (43). Although daily administration of BNZ is important to achieve the trypanocidal effect, it also increases the concentrations of toxic BNZ metabolites, the major drawback of current BNZ therapy (19). Because BNZ is practically insoluble in water (50), its slow release from the PSs to the aqueous environment of the cytoplasm was likely responsible for the successful antiparasitic effect of only 2 BNZ-PS administrations during the 14 days of treatment. An equally important finding from our study is that the low effective dose of BNZ achieved through the BNZ-PS formulation completely abrogated the weight loss and hepatotoxicity observed with the effective dose of free-form BNZ.

In conclusion, we have developed an advanced formulation of BNZ based on incorporation into a PEG-*b*-PPS nanocarrier that increased the potency and decreased the toxicity of this effective trypanocidal drug. The likely mechanisms were enhanced cytoplasmic delivery via well-established uptake mechanisms and reduced exposure of BNZ and its metabolites due to molecular sequestration within PS nanocarriers. The most clinically relevant consequence of BNZ-PS was the reduction of cardiac parasitosis and inflammation, which strongly supports further development of PEG-*b*-PPS nanocarriers for Chagas drugs and drug combinations. Ultimately, a sustained-release BNZ formulation may further advance therapy. PEG-*b*-PPS nanocarriers have been employed for sustained intracellular drug release for up to 12 days through the modification of the PPS chain to achieve slow release by partitioning the drug from the hydrophobic PPS phase into the aqueous phase (35, 51, 52) and PS can also be delivered by routes other than i.v. (53, 54). Furthermore, PEG-*b*-PPS nanostructures can be surface-engineered for cell selective receptor-mediated uptake (29, 30, 33). These approaches could also be of use in treating other intracellular parasitic, viral, bacterial, and fungal infections, which are often treated with highly toxic drugs.

## Methods

### Synthesis of PEG-b-PPS copolymers and loading of BNZ into PSs.

PSs were prepared by the controlled self-assembly of PEG-*b*-PPS block copolymers with the 25%–45% molecular weight of hydrophilic PEG fraction in the total block copolymer. PEG-*b*-PPS block copolymers were synthesized as previously described (30). Briefly, the anionic ring-opening polymerization of propylene sulfide was initiated by PEG thioacetate and end-capped with PEG mesylate. The obtained block copolymers (PEG_17_-PPS_60_-PEG_17_) were purified by precipitation in methanol and then characterized by NMR spectroscopy and gel permeation chromatography (Thermo Fisher Scientific). The loading of BNZ (MilliporeSigma) into PS to make BNZ-PS was performed by the thin-film rehydration method in PBS as described previously (30, 39). Briefly, 30 mg of the copolymer (PEG_17_-PPS_60_-PEG_17_) with or without 1.5 mg of BNZ was dissolved in 150 μL tetrahydrofuran within 1.8 mL clear glass vials (Thermo Fisher Scientific) and placed under vacuum to remove the solvent. The resulting thin films were dehydrated in sterile PBS (1 mL) under shaking at 1500 rpm for 48 hours. The BNZ-PSs were purified to remove free BNZ by Zeba Spin Desalting Columns (7K MWCO, Thermo Fisher Scientific).

### Characterization of PSs.

LC-MS was performed using a Bruker AmaZon-X instrument. Samples were chromatographed on a Hypersil BDS C18 column (2.4 mM particle size, 2.1 × 50 mM) (Thermo Fisher Scientific) at 40°C. The separation was achieved by a gradient of water with 0.1% formic acid (eluent A) and acetonitrile with 0.1% formic acid (eluent B) with a flow rate of 0.3 mL/min. Detection was performed at 324 nm and the injection volume was 2 μL. The gradient was started at 40% B for 1 minute, increased to 100% B over 4 minutes, held at 100% B for 5 minutes, decreased to 90% B over 0.1 minute, and held at 90% B for 1.9 minutes. The standard calibration solution of BNZ was prepared in acetonitrile/water (95:5 v/v), ranging from 3.125 to 100 mg/mL. BNZ-PS samples were dissolved in acetonitrile/water (95:5 v/v) and then filtered through a 0.2 mM membrane (Thermo Fisher Scientific). The loading efficiency of the BNZ-PS was determined by the percentage of the loaded weight of BNZ of the total weight of BNZ-PS. The encapsulation efficiency of the BNZ-PS was calculated by the percentage of BNZ weight loaded into the PS of the initial BNZ weight used. The size distribution and zeta potential of PS and BNZ-PS (1 mg/mL) were characterized by Zetasizer Nano-ZS using a 4 mW He-Ne 633 laser (Malvern Instruments). The morphology of PS and BNZ-PS was determined by cryo–transmission electron microscopy as described previously (55). In brief, 200 mesh Cu grids with a lacey carbon membrane (catalog LC200-CU-100, EMS) were glow discharged in a Pelco easiGlow glow discharger (Ted Pella Inc.) using an atmosphere plasma generated at 15 mA for 15 seconds with a pressure of 0.24 mbar. PS and BNZ-PS samples (4 μL, 10 mg/mL in PBS) were pipetted onto the grid and blotted for 5 seconds with a blot offset of +0.5 mM, followed by immediate plunging into liquid ethane within an FEI Vitrobot Mark III plunge-freezing instrument (Thermo Fisher Scientific). The plunge-frozen grids were kept vitreous at –180°C in a Gatan Cryo Transfer Holder model 626.6 (Gatan Inc.) and viewed in a JEOL JEM1230 LaB6 emission transmission electron microscope (JEOL USA, Inc.) at 100 kiloelectron volts. Image data were collected by a Gatan Orius SC1000 CCD camera model 831 (Gatan Inc.). The images were processed and analyzed using NIH ImageJ software. To test the stability of BNZ-PSs upon storage, a suspension of BNZ-PSs (30 mg/mL in PBS) was kept in sealed tubes and maintained at 4°C. At different time points (0, 1, 4, 8, 15, and 45 days), the released or unloaded BNZ was removed by using Zeba Spin Desalting Columns (7K MWCO, Thermo Fisher Scientific). The percentage of loaded BNZ in PS at different time points (compared with day 0) was determined by LC-MS by methods mentioned above.

### Cells, cell culture, and parasite purification.

The Y strain of *T*. *cruzi* was used for all experiments other than the nanocarrier uptake/cell imaging experiment, which employed Tulahuen strain expressing the dual reporter mNeonGreen fused to red-shifted luciferase (Luc-mNeonGreen) (56). Epimastigotes were cultured using standard methods. H9C2 rat myoblasts (ATCC) were inoculated with small numbers of cultured log-phase epimastigotes, which contain small numbers of metacyclic trypomastigotes capable of initiating mammalian cell infection. After several days of culture in H9C2 cells, amastigotes and trypomastigotes are produced, and trypomastigotes can be purified from culture supernatants using standard methods. Amastigotes can be easily studied in situ as intracellular parasites (e.g., IC_50_ determination) or purified from host cells for additional studies.

### Intracellular amastigotes.

H9C2 rat myoblast cells, 50,000 per well, were seeded in Chamber Slides (Nunc Lab-Tek II, Thermo Fisher Scientific) and incubated in RPMI medium (RPMI 1640, 5% FBS, 100 IU/mL penicillin, 100 μg/mL streptomycin, and 2 mM l-glutamine) at 37°C in 5% CO_2_. After 24 hours, cells were infected by the addition of 250,000 *T*. *cruzi* Y strain trypomastigotes per well (5:1 MOI), incubated for 24 hours, and washed to remove trypomastigotes in the medium. Different concentrations of BNZ and BNZ-PS in RPMI were added to each well. The medium (with drug) was replaced after 24 hours, and the slides were fixed and stained with Giemsa after a further 24-hour incubation. The number of amastigotes per 100 H9C2 cells was calculated for each chamber. The Resazurin method was used to evaluate the activity of BNZ and BNZ-PS against trypomastigotes. Purified trypomastigotes (1 × 10^7^ cells/mL) were incubated in 96-well plates containing serial dilutions of BNZ or BNZ-PS in RPMI medium. After incubation for 24 hours, 10 μL of alamarBlue cell viability reagent (Thermo Fisher Scientific) was added to each well, and plates were incubated for an additional 4 hours. Fluorescence was measured on a microplate reader (BMG Labtech) at 560/590 nm to evaluate trypomastigote viability. A log concentration versus response curve was generated, and the BNZ IC_50_ was calculated using GraphPad Prism 7.0.

### Nanocarrier uptake.

H9C2 cells were seeded in Chamber Slides for 24 hours and then infected with Luc-mNeonGreen–expressing *T*. *cruzi* trypomastigotes at a 5:1 MOI for 24 hours. Free trypomastigotes were washed away and the *T*. *cruzi*–infected H9C2 cells were incubated with Alexa Fluor 630–labeled PS for 24 hours. The slides were washed 5 times with PBS and stained with CellMask Deep Red Plasma Membrane Stain (Invitrogen, Thermo Fisher Scientific) for 5 minutes and DAPI for 20 minutes. Leica confocal microscopy was used to image the slides.

### Mouse infections.

Female BALB/c mice (4–6 weeks of age) were purchased from The Jackson Laboratory and housed in pathogen-free conditions on a 12-hour dark/12-hour light cycle at 22 ± 3°C with access to food and water ad libitum. H9C2-derived *T*. *cruzi* Y strain tissue culture trypomastigotes to be used for infections of BALB/c mice were first passaged through female SCID mice (The Jackson Laboratory) bloodstream trypomastigotes. SCID mice were infected by i.p. injection of 2 × 10^3^ tissue culture trypomastigotes in 0.2 mL PBS and peripheral blood parasitemia was monitored by analysis of saphenous venous blood (3 μL). When SCID mice parasitemia reached 1 × 10^8^ trypomastigotes/mL, infected blood from SCID mice was harvested and adjusted to 2 × 10^4^ bloodstream trypomastigotes/mL by the addition of PBS. BALB/c mice were then infected with 2 × 10^3^ bloodstream trypomastigotes by i.p. injection. Parasitemia of Y strain–infected mice was determined and animals were euthanized after the final time point.

### BNZ and BNZ-PS therapy.

BNZ-PS was prepared as described above, and BNZ (MilliporeSigma) was prepared from powder at 22 mg/mL in 5% methylcellulose. After confirming establishment of infection by peripheral blood analysis 7 d.p.i., 30 mice were randomized into 6 groups of 5 animals each, as follows: (a) untreated control mice, (b) mice administered 0.3 mg/mL PS by i.v. injection, (c) mice treated with 100 mg/kg BNZ daily orally (po) for 14 days (7–21 d.p.i.), (d) mice treated with BNZ-PS i.v. at a BNZ dose of 1.5 mg/kg 7 and 14 d.p.i. (2 doses), (e) mice treated with BNZ-PS i.v. at a BNZ dose of 0.15 mg/kg 7 and 14 d.p.i. (2 doses), and (f) mice treated with BNZ-PS at a BNZ dose of 0.03 mg/kg 7 and 14 d.p.i. (2 doses). All animals were euthanized 25 d.p.i. and blood and organs were procured for further analysis.

### Tissue parasitosis.

Organs were harvested and snap-frozen on dry ice. DNA was extracted using QIAamp DNA Mini Kit (QIAGEN) following the manufacturer’s instructions. To make the standard for parasite burden quantification, 25 mg of heart of noninfected mouse was mixed with 1 × 10^7^ *T*. *cruzi* trypomastigotes. Total DNA was extracted, and the DNA concentration was adjusted to 50 ng/μL. The standard curve was established from serial dilutions of the sample, ranging from 1 × 10^7^ to 1 × 10^–1^ parasite equivalents. Real-time PCR reactions were carried out using a QuantStudio 5 instrument (Thermo Fisher Scientific). PCR reactions contained 50 ng of DNA, 0.5 μL of primers TCZ-F 5′-GCTCTTGCCCACAMGGGTGC-3′ and TCZ-R 5′-CCAAGCAGCGGATAGTTCAGG-3′, and 10 L of EXPRESS SYBR GreenER qPCR Supermix (Invitrogen, Thermo Fisher Scientific) in a final volume of 20 L. Reactions were run in triplicate using the following cycling parameters: 50°C for 2 minutes followed by 40 cycles of 95°C for 10 seconds, 55°C for 15 seconds, 72°C for 5 seconds. Mouse-specific GADPH forward and reverse were used as internal controls.

### Histopathology and image analysis.

Hearts were obtained from all mice, fixed in 10% buffered formalin for 15 hours, and embedded in paraffin. Five-micrometer sections were stained with H&E and Masson’s trichrome stain. All slides were scanned using an Aperio Scanscope AT slide scanner, and images were taken with ImageScope software and analyzed using NIH ImageJ software by a scientist blinded to the groups. The inflammatory index was derived by quantifying the total number of nuclei present in 10 randomly selected microscopic fields of each H&E-stained section. Tissue cellularity and percentage of cellular area occupied by nuclei were determined as separate but related histologic indicators of inflammation.

### Assessment of the toxicity of BNZ and BNZ-PS in vivo.

Twenty-five healthy BALB/c mice were randomized into 5 groups of 5 animals each, as follows: (a) untreated control mice, (b) mice administered 0.3 mg/mL PS i.v., (c) mice treated with 100 mg/kg BNZ daily po for 14 days (days 7–21), (d) mice treated with BNZ-PS i.v. at a BNZ dose of 1.5 mg/kg on days 7 and 14 (2 doses), and (e) mice treated with BNZ-PS i.v. at a BNZ dose of 0.15 mg/kg on days 7 and 14 (2 doses). Mouse weights were recorded during the course of the experiment. On day 15, mice were euthanized and serum and liver tissue were collected from each animal. Serum alanine aminotransferase (ALT/SGPT), was determined at a reference laboratory (IDEXX BioResearch).

### Statistics.

Numerical results are expressed as mean ± SEM. Individual animals were used as the unit of analysis for in vivo and ex vivo experiments. Animal group size was determined empirically. One-way ANOVA and Tukey’s multiple comparisons test were used in GraphPad Prism v.7 to evaluate differences between groups. *P* values of less than 0.05 were considered statistically significant.

### Study approval.

All animal protocols were reviewed and approved by the Institutional Animal Care and Use Committee of Cedars-Sinai Medical Center (protocol 7053).

## Author contributions

XL, SY, EAS, and DME conceived and designed the research. XL, SY, SJM, BAF, and CLO carried out all the experiments and analyzed the data. XL, SY, DBS, PSR, EAS, and DME wrote the manuscript with feedback from all the authors. XL is listed before co–first author SY because she did more than half of the work on this project.

## Figures and Tables

**Table 1:**
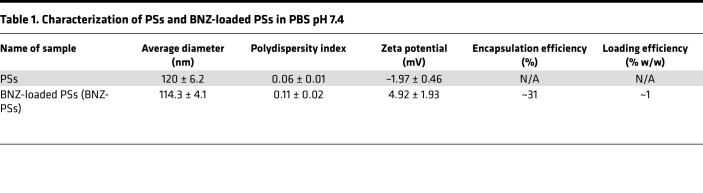
Characterization of PSs and BNZ-loaded PSs in PBS pH 7.4

## Version 1 05/10/2021

Electronic publication
